# Effect of *Elaeagnus angustifolia* Linn. on the Physicochemical Properties and Microbial Community Structure of Inter-Rhizosphere Soils

**DOI:** 10.3390/plants14081242

**Published:** 2025-04-18

**Authors:** Mengyi Sui, Xin Qin, Nan Sun, Yangbo Liu, Chen Yang, Luofei Guan, Yawen Zhang, Haiyan Wang, Manman Zhang, Yunfei Mao, Xiang Shen

**Affiliations:** 1 College of Horticultural Science and Engineering, National Apple Engineering and Technology Research Center, Shandong Agricultural University, Tai’an 271002, China; smy09042000@163.com (M.S.); qinxinsdau@163.com (X.Q.); liuboyangsdau@163.com (Y.L.); 2021120417@sdau.edu.cn (C.Y.); glf199649@163.com (L.G.); zyw18905363662@163.com (Y.Z.); zhangmanman@sdau.edu.cn (M.Z.); 2 College of Horticulture, Hebei Agricultural University, Baoding 071051, China; 2021120397@sdau.edu.cn; 3 College of Forestry, Shandong Agricultural University, Tai’an 271002, China; haiyanwang@sdau.edu.cn

**Keywords:** inter-rooted soils of *E. angustifolia* L., properties of soil in terms of physics and chemistry, high-throughput sequencing, soil microbial community structure

## Abstract

Aims: The aim of this study was to elucidate the effect of *Elaeagnus angustifolia* Linn. (*E. angustifolia* L.) on the structure and abundance of the soil microbial community. This paper provides a theoretical foundation for guiding the establishment of *E. angustifolia* L. forests to enhance the physicochemical properties of soil. Methods: This study employed high-throughput sequencing technology to analyse the composition, diversity, and structural changes of various soil fungal and bacterial communities and correlated the results with soil physicochemical properties. Results: The results indicated a significant increase in the total nitrogen (0.66 g/kg–0.87 g/kg), ammonium nitrogen (3.60 mg/kg–6.56 mg/kg), and organic matter (1.06–1.38%) contents of the inter-rhizosphere soil of *E. angustifolia* L. after 3, 4, and 5 months of planting. Additionally, the total phosphorus, potassium, and nitrate nitrogen contents increased, whereas soil pH and salinity decreased. The abundance of soil microbial communities also increased. The fungal phyla with relative abundances greater than 1% were *Ascomycota*, Fungi_unclassified, *Basidiomycota*, *Zygomycota*, and *Glomeromycota*. *Chytridiomycota*, *Rozellomycota*, *Mortierellomycota*, and *Olpidiomycota* were not found in the bare soil control but were observed in the rhizosphere soil of the date palm. The relative abundance of bacteria from the phyla *Proteobacteria*, *Acidobacteria*, *Actinobacteria*, *Gemmatimonadetes*, and *Chloroflexi* in the inter-root soil of jujube dates showed an increase in comparison with the control group. At the same time, correlation analysis found that soil total phosphorus, nitrogen content, and soil enzyme activity were positively correlated with the bacterial level, with TN (*p* < 0.01) and NO_3_^−^-N (*p* < 0.05) showing significant positive correlations. Conversely, soil pH and salinity were mostly negatively correlated with the fungi, and soil enzyme activity was significantly correlated with the fungal and bacterial at different RAD levels. Conclusions: The introduction of *E. angustifolia* L. markedly affected the physicochemical properties and microbial community composition of the soil.

## 1. Introduction

The intricate interplay between material and energy transfer among plants, soil, and microbes forms a complex network of interactions. Vegetation cover is instrumental in promoting the growth, development, and reproduction of microorganisms by supplying energy and creating a favourable environment. These interactions can significantly affect the composition and size of the microbial communities [1]. In recent years, China has encountered substantial challenges in forestry and agricultural growth due to large-scale environmental issues, such as soil salinisation and land desertification. Numerous studies have demonstrated that the structure and abundance of soil microbial communities can be modified by vegetation cover and tillage planting. This modification improves soil structure and physicochemical properties, thereby mitigating environmental problems, such as soil salinisation and land desertification [2].

Soil microorganisms, characterised by their vast numbers and complex interactions, are involved in the ecological regulation of the soil microenvironment. They are crucial drivers of soil nutrient cycling, with significant ecological regulatory functions [3], whereas plant diversity is a primary factor that controls constant changes in soil microorganisms [4]. Lyu et al. discovered that Simpson’s index of understory plant diversity and soil bacterial α-diversity could be significantly increased by enlarging the gaps in plantation forests of *Picea abies*. They determined that plant diversity is associated with soil bacterial communities [5]. Moreover, soil microbial abundance and the activity of certain enzymes involved in C and N cycling significantly increase with ground cover plant diversity [6]. According to Shao et al., the introduction of artichoke cultivation resulted in a notable increase in the abundance and diversity of soil microbial communities, thereby improving soil fertility [7]. The abundance and diversity of the soil bacterial communities increase with the duration of *E. angustifolia* L. cultivation. Notably, the dominant phylum is Ascomycota, whereas the dominant genus is *Pseudomonas*. *Pseudomonas* plays a crucial role in the synthesis of key soil enzymes that are essential regulators of carbon and nitrogen dynamics [8]. Simultaneously, it has been discovered that *E. angustifolia* L. forms a symbiotic relationship with various nitrogen-fixing actinobacteria of the genus *Frankia*. This symbiosis is beneficial to *E. angustifolia* L., as these Actinobacteria convert N_2_ into a fixed form that can be readily absorbed and utilised by the plant. *Frankia* is a salt-tolerant bacterium that, after symbiosis with the root system of *E. angustifolia* L., improves its adaptability to saline and alkaline soils [9]. Several studies have shown that cultivation of *E. angustifolia* L. can significantly enhance the levels of total nitrogen, effective phosphorus, and organic nitrogen in the soil. Additionally, this cultivation method has been found to augment soil enzyme activity and ameliorate soil infertility [10]. Moreover, *E. angustifolia* L. can coexist with microorganisms to produce rhizomes that improve soil fertility and fix atmospheric nitrogen using the roots and mycelium [11].

*E. angustifolia* L. is a salinity-tolerant multipurpose tree species that has demonstrated significant ecological utility in the reforestation of saline and sandy lands [12]. Although *E. angustifolia* L. has demonstrated ecological utility in the reforestation of saline and sandy soils, there is a lack of comprehensive studies specifically addressing its impact on soil ecosystems. This study focuses on the unique characteristics and impacts of *E. angustifolia* L. to fill this gap by providing detailed information on how it alters soil microbial communities, soil physicochemical properties, and soil enzyme activities. Although general studies on the relationship between vegetation and soil microbial communities already exist, this study was conducted specifically on *E. angustifolia* L. It specifically examined changes in the physicochemical properties and microbial communities of inter-root soils of the species as well as soils without the species. The detailed investigation of this particular tree species contributes to a deeper understanding of its ecological functions and provides a theoretical basis for guiding the establishment of *E. angustifolia* L. forests to improve the physicochemical properties of soil.

## 2. Materials and Methods

### 2.1. Experimental Area and Cultivation of Test Materials

The experimental site was located in Li Jia Dapo Village, Ma Zhuang Town, Daiyue District, Tai’an City, Shandong Province, in China. The park was transformed from local wheat-growing land. The coordinates are 36°01′11″ N, 117°0′11″ E. The region experiences a warm, temperate, and semi-humid monsoon climate [13]. The soil type was haplic solonchaks, 25 cm thick salt deposit, ECe = 5–8 dS/m, frost-like and weakly massive structures, <20% water-stable aggregates, pH 6.8–7.5, organic matter content 0.8–1.2%, and cation exchange 8–12 cmol(+)/kg.

Fifty fruits of *E. angustifolia* L. were collected and soaked in edible water for 72 h. The flesh was then rubbed off and the seeds were air-dried under ventilated conditions. The seeds were mixed with wet sand in woven bags and placed outdoors in a sheltered area for stratification. In March 2021, the sowing process commenced once two-thirds of the seeds turned white. A small arch was established, and the ground was prepared by ensuring that it was well watered. The seeds were sowed and covered with 1 cm of soil. During this period, it was crucial to maintain adequate heat preservation and ventilation. Watering was conducted every 15 days to maintain soil moisture at 60–70%, promoting shoot germination. After 25 d, the resistance of the seedlings was exercised. Throughout the growth period, temperature was maintained at 15–28 °C, with a daily light exposure of 10 h, and watering was performed every 20 days. After 40 d, the seedlings were ready to be transplanted into the field.

Soil sampling was conducted during the summer of 2021. Ten well-growing *E. angustifolia* L. plants were selected in mid-July, mid-August, and mid-September, and five plants were randomly selected using the S-shaped sampling method. The roots of the plants were gently shaken, and the soil tightly adhering to the roots was collected as rhizosphere soil. Any gravel and crop roots were removed using sterilised forceps. The inter-root soil from the *E. angustifolia* L. was then placed in sterile sealing bags and securely sealed [14]. Simultaneously, five sampling points were selected along the S-shape outside the projection of the date palm in the planting area to collect 0–20 cm soil samples as non-inter-root soil. The soil was mixed at each sampling point and recorded as 7 W, 8 W, and 9 W for each month. Each treatment consisted of three independent replicates. The collected soil was immediately transferred to a portable icebox for transportation back to the laboratory. Upon arrival at the laboratory, the inter-root matrix was sieved through a 2 mm sieve to remove stones and roots. A part of the soil was naturally dried and ground for soil physicochemical property determination, while the remainder was stored in a refrigerator at −80 °C for soil microbial sequencing.

### 2.2. Test Methods

Soil samples were determined by decocting the prepared soil samples using the combined H_2_SO_4_-H_2_O_2_ decoction method. Soil total nitrogen was determined using a multi N/C 3100 (Analytik Jena AG, Jena, Germany) analyser. Soil total phosphorus was determined using the molybdenum blue colorimetric method, and soil total potassium was determined using flame spectrophotometry [15]. Soil ammonium nitrogen and nitrate nitrogen contents were determined using UV spectrophotometry [16]. Each treatment consisted of three biological replicates.

The determination of soil-resistant enzymes was conducted as follows: urease was determined using the colorimetric method, catalase was determined using the volumetric method, sucrase was determined using the colorimetric method, and phosphatase was determined using the colorimetric method. The soil salt content was determined using a DDS-307A conductivity meter (Shanghai Yidian Scientific Instrument Co., Ltd., Shanghai, China), and the soil pH was determined using a pH meter FE28-Standard(METTLER TOLEDO, Shanghai, China) [17,18]. Soil organic matter was determined using the hydrated thermal potassium dichromate oxidation colorimetric technique described by Okalebo et al. [19]. Each treatment was replicated thrice to ensure biological consistency.

### 2.3. Soil DNA Extraction and High-Throughput Sequencing

Total deoxyribonucleic acid (DNA) was extracted using the FastDNA^®^ SPIN Kit for Soil (Norcross, MP, USA). The quality of the extracted DNA was assessed by 1% agarose gel electrophoresis. Universal primers were utilised based on the conserved regions in the sequence, with 5–50 ng of DNA serving as the template. The primers for the V3–V4 region of the bacterial 16sRNA gene were 341F (5′-CCTACGGGNGGCWGCAG-3’) and 805R (5′- GACTACHVGGGTATCTAATCC-3′) [20]; the primers for the ITS2 region of the fungal 18S rRNA gene were fITS7 (5′-GTGARTCATCGAATCTTTG-3′) and ITS4 (5′-TCCTCCGCTTATTGATATGC-3′) [21]. The libraries underwent an initial quality assessment. Subsequently, only those that met the quality standards were selected for sequencing. The IlluminaHiSeq2500 platform (Shandong Senqi Biotechnology Co., Ltd., Jinan, China) was used to obtain high-throughput sequencing data for the soil planted with *E. angustifolia* L. (E) and the soil without *E. angustifolia* L. (W) [22].

After the up-sequencing was completed, we obtained the raw down-sequencing data, used overlapping to splice the double-ended data, and performed quality control and chimera filtering to obtain high-quality clean data. DADA2 (Divisive Amplicon Denoising Algorithm) was used through dereplication and other steps, and then, DADA2 obtained representative sequences with single-base precision and used the concept of ASVs (amplicon sequence variants) to construct an OTU (operational taxonomic unit) table to obtain the final feature character table as well as the feature sequences and further carry out the diversity analysis, species taxonomic annotations, and variance analysis. Based on the feature abundance table, we performed a PCA analysis using the vegan package in R programming language, where the more similar the species composition of the samples, the closer they are in the PCA plot. Based on the results of the feature analysis of each sample, we used two indicators, unweighted unifrac and weighted unifrac, to measure the dissimilarity between two samples. The smaller the value, the smaller the difference between these two samples in terms of species diversity. The feature sequences are the raw files for our species classification; in order to analyse the species composition more accurately, we used SILVA (Release 132, https://www.arb-silva.de/documentation/release-132/, accessed on 7 July 2022), the NT-16S database, RDP, and the UNITE database for species classification and subsequent analyses to ensure complete and accurate annotation results. Based on the results of feature annotation and the feature list of each sample, we obtained the species abundance table at the level of kingdom, phylum, order, family, genus, and species and carried out the species composition and difference analysis of different samples (groups) for different levels of the species abundance table with the following parameter threshold: confidence level > 0.7. A cluster analysis was performed on the samples based on the distance of the species composition of the samples. The analysis was implemented using the vegan package in R programming language, and samples were clustered using the Bray–Curtis distance (one of the most commonly used distance metrics in systematic clustering methods, mainly used to describe the degree of proximity between samples, and the magnitude of the distance is the main basis for the classification of the samples). The RDA analyses were carried out using the vegan package in R programming language for PCA to obtain the unbound ordination axes (principal components). Environmental variables were used as constraints, and the constrained ordination axes most relevant to the species data were filtered by multiple regression fitting so that the variance of the axes was explained as much as possible by the environmental variables. The permutation test was used to determine whether the environmental variables significantly affected species composition. Significance tests were conducted for each environmental variable using anova.cca() from the vegan package in R programming language, combined with F-values and permutation tests *p*-values to screen for key factors. Finally, the Shapiro–Wilk test was used to check whether the residuals conformed to normal distribution and the chi-square test.

### 2.4. Data Processing

The experimental data were initially processed and analysed visually using Microsoft Excel 2021 software, including the data collation, calculation of basic statistics, and graph drawing. SPSS 22.0 software was applied in the statistical analysis section to carry out LSD and an independent samples t-test, which were used to assess the significance of the difference in means between the two groups, respectively. All statistical tests were performed with *p* < 0.05 as the significance threshold and *p* < 0.01 as the highly significant threshold to ensure the reliability of the results. High-throughput sequencing data were analysed online using the Lianchuan BioCloud platform (https://www.omicstudio.cn/home, accessed on 17 July 2022).

## 3. Results

### 3.1. Effect of E. angustifolia *L.* on Soil Physicochemical Properties

#### 3.1.1. Effect of *E. angustifolia* L. on Soil Nutrients

**Figure 1:**
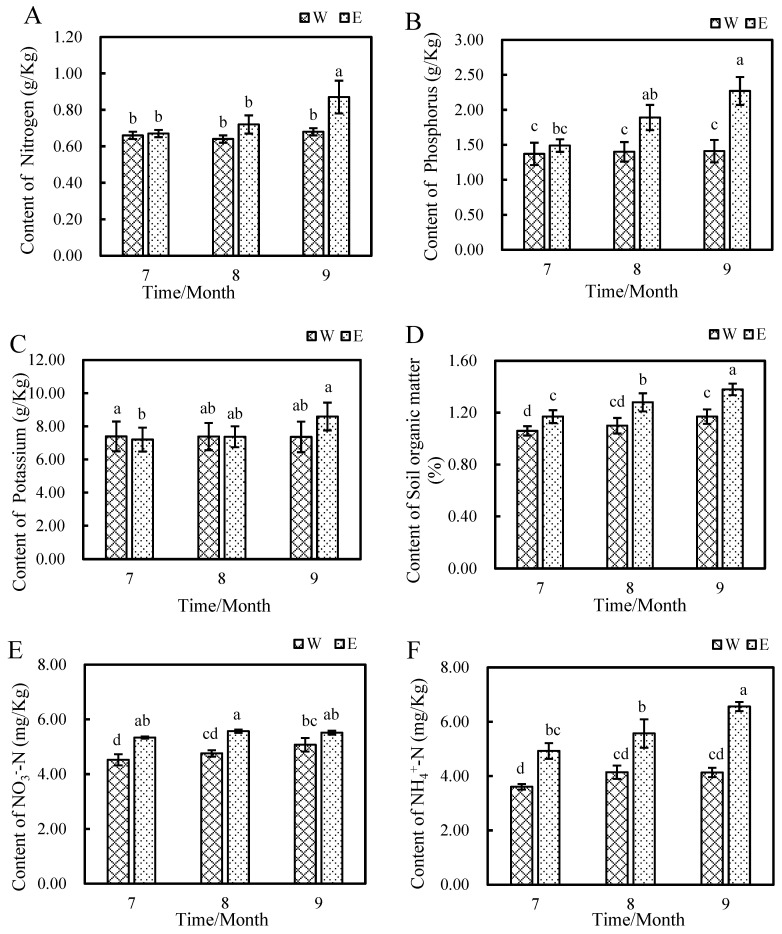
Changes in rhizosphere soil nutrient contents between W and E from July to September. (**A**) Nitrogen content; (**B**) phosphorus content; (**C**) potassium content; (**D**) soil organic matter content; (**E**) NO_3_^−^-N content; (**F**) NH_4_^−^-N content. Note: W: Uncultivated soil with *E. angustifolia* L.; E: soil for planting *E. angustifolia* L. Error bars represent the standard error of the mean (n = 3). Different letters above the columns indicate significant differences according to the LSD test (*p* < 0.05).

Figure 1 illustrates the nutritional composition of the soil within the root system of *E*. *angustifolia* L. The contents of total nitrogen (TN), nitrate nitrogen (NO_3_^−^-N), and ammonium nitrogen (NH_4_^+^-N) exhibited a consistent upward trend in both the soil where *E*. *angustifolia* L. was planted and the soil where it was not planted, reaching their peaks in September. In the soil for planting *E*. *angustifolia* L., the TN content increased by 31.81%, the nitrate nitrogen rose from 4.53 mg/kg to 5.52 mg/kg, and the ammonium nitrogen also increased from 3.60 mg/kg to 6.56 mg/kg. In August and September, there were significant differences in the soil total phosphorus (TP) between the two types of soil. The soil for planting *E*. *angustifolia* L. was 0.49 g/Kg and 0.86 g/Kg higher than the unplanted soil, respectively, and the content reached the highest value in September. There was no significant difference in the total potassium (TK) content between the two types of soil. In the soil where *E. angustifolia* L. was planted, the content increased from 7.2 g/Kg to 8.59 g/Kg, reaching the highest value in September, while in the unplanted soil, it decreased from 7.39 g/Kg to 7.36 g/Kg. TN and TP are readily absorbed by plants to support their growth. The content of soil organic matter in the soil where *E. angustifolia* L. was planted was significantly higher than that in the unplanted soil in July, August, and September, being 0.11%, 0.18%, and 0.21% higher, respectively.

#### 3.1.2. Effect of *E. angustifolia* L. on Soil Salinity and pH

**Figure 2:**
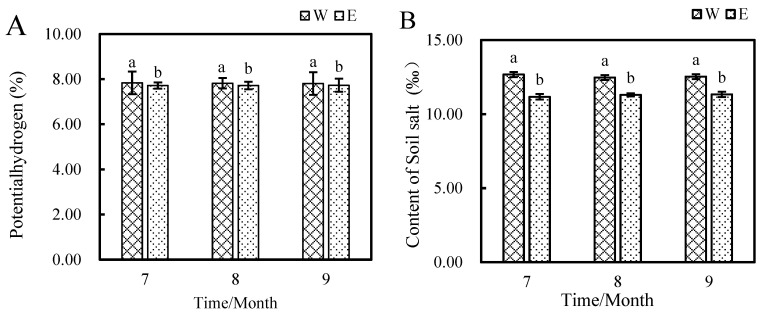
Rhizosphere soil (in water) pH and salt concentration in W and E from July to September. (**A**) Potential hydrogen of soil; (**B**) soil salt content. (‰ is a thousandth of a cent.) Note: W: Uncultivated soil with *E. angustifolia* L.; E: soil for planting *E. angustifolia* L. Error bars represent the standard error of the mean (n = 3). Different letters above the columns indicate significant differences according to the LSD test (*p* < 0.05).

Figure 2 shows the soil pH and salinity of *E. angustifolia* L. Soil pH decreased from 7.84 to 7.73 from July to September, representing a decrease of 1.42%. The salinity of the soil where *E. angustifolia* L. was planted showed a decreasing trend each month for three months compared with that of the control, dropping from 12.67‰ to 11.33‰.

#### 3.1.3. Effect of *E. angustifolia* L. on Soil Enzyme Activities

**Figure 3:**
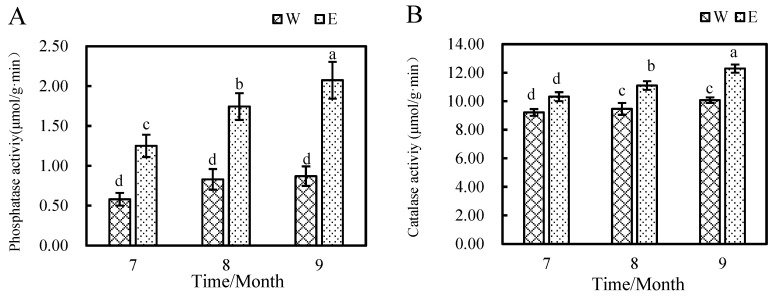

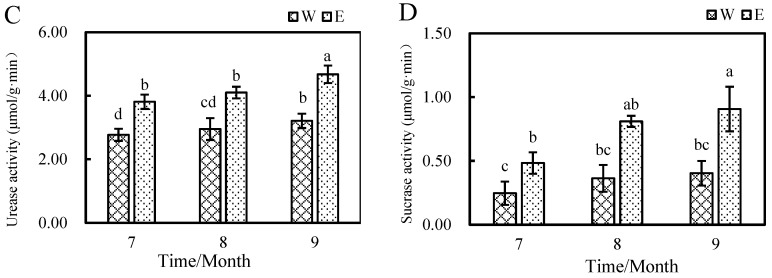
Changes in enzyme activities in rhizosphere soil between W and E from July to September. (**A**) Soil phosphatase activity; (**B**) catalase activity; (**C**) soil urease activity; (**D**) soil sucrase activity. Note: W: Uncultivated soil with *E. angustifolia* L.; E: soil for planting *E. angustifolia* L. Error bars represent the standard error of the mean (n = 3). Different letters above the columns indicate significant differences according to the LSD test (*p* < 0.05).

The results of the enzyme activity test of the inter-rhizosphere soil of *E. angustifolia* L. are shown in Figure 3. After planting *E. angustifolia* L., the activities of four enzymes (sucrase, phosphatase, urease, and catalase) in the inter-rhizosphere soil of *E. angustifolia* L. gradually increased from July to September, reaching their maximum values in September. Among these, the activity of sucrase increased the most, from 0.25 μmol/g·min to 0.91 μmol/g·min, an increase of 267.53%. Sucrase can hydrolyse sucrose, thereby improving soil nutrients. Next, phosphatase activity increased from 0.58 μmol/g·min to 2.07 μmol/g·min, which represented an increase of 256.90%. Phosphatase can dephosphorylate and produce phosphate ions. Its activity increased with increasing planting time, which had a certain effect on the soil total phosphorus content. Urease activity increased by 68.71%, and catalase activity increased by 33.30% from 9.22 μmol/g·min to 12.29 μmol/g·min.

### 3.2. High-Throughput Sequencing Analysis of Inter-Rhizosphere Soil of E. angustifolia L.

**Figure 4:**
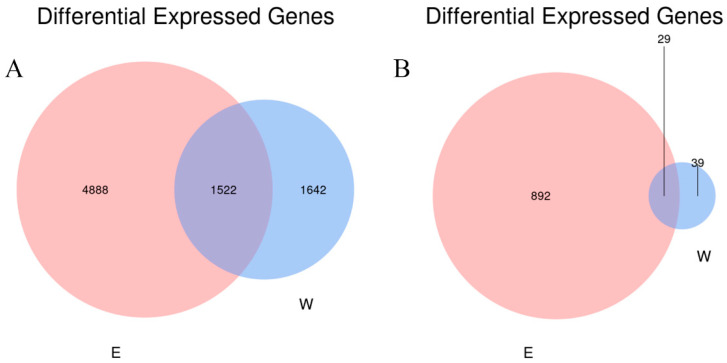
Venn diagram of OTUs in W and E. (**A**) Venn diagram of bacterial OTUs; (**B**) Venn diagram of OTUs fungi. Note: W: Uncultivated soil with *E. angustifolia* L.; E: soil for planting *E. angustifolia* L.

The number of operational taxonomic units (OTUs) for bacteria and fungi were obtained by QC and filtering at a 97% sequence similarity level. As shown in Figure 4, there are 6410 bacterial OTUs in the rhizosphere soil samples and 3164 in the non-rhizosphere soil samples. Among them, 1522 are the same in both. There are 4888 unique bacterial OTUs in the rhizosphere soil samples, which are 3246 more than those in the non-rhizosphere soil samples. There are 921 fungal OTUs in the rhizosphere soil samples and 68 in the non-rhizosphere soil samples. There are 29 common fungal OTUs in both. There are 892 distinctive fungal OTUs in the rhizosphere soil samples, which are 853 more than those in the non-rhizosphere soil samples. As depicted in Supplementary Figure S1, the rarefaction curve for bacteria and fungi gradually increased and levelled off with the number of samples taken. Whether it is bacteria or fungi, the numbers are substantially increased in the rhizosphere soil, indicating that the cultivation of *E. angustifolia* L. has increased the types of soil microorganisms.

### 3.3. Effect of E. angustifolia *L.* on the Composition of Soil Microbial Communities

#### 3.3.1. Soil Bacterial Communities Composition

**Figure 5:**
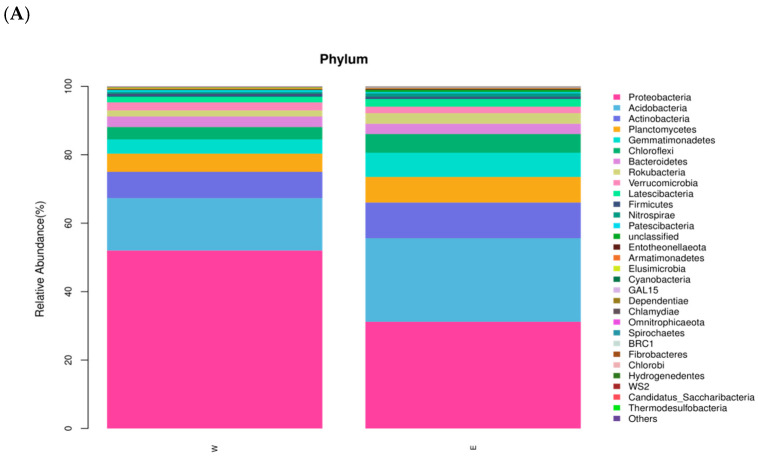

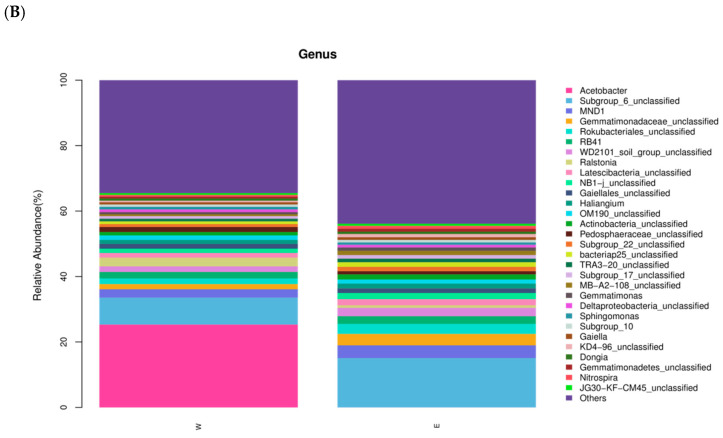
Abundance map of bacteria communities at the phylum level (**A**) and genus level (**B**) in W and E. Note: W: Uncultivated soil with *E. angustifolia* L.; E: soil for planting *E. angustifolia* L.

Figure 5A illustrates the composition of the soil bacterial communities at the phylum level, highlighting the top 30 taxa in terms of their relative abundance. The top 10 phyla in relative abundance were *Proteobacteria*, *Acidobacteria*, *Actinobacteria*, *Planctomycetes*, *Gemmatimonadetes*, *Chloroflexi*, *Bacteroides*, *Rokubacteria*, *Verrucomicrobia*, and *Latescibacteria*. The comparative prevalence of seven phyla in the rhizosphere soil of *E. angustifolia* L. was identified to be higher than that in the bare soil. The relative abundance of *Proteobacteria* in all of the samples was higher than 25%, making it the dominant phylum. However, the relative abundance of *Proteobacteria* in the rhizosphere soil of *E. angustifolia* L. was 31.17% compared with that of the control. The abundance of *Proteobacteria* in the control was rapidly increased by other phyla, leading to a significant increase in fungal abundance in the rhizosphere soil of *E. angustifolia* L. The relative abundance of *Bacteroides* was also lower than that of the control because the soil microenvironment was not restored in the short planting period. The relative abundance of *Acidobacteria* was 24.45%, which was 9.10% higher than that in the control. The relative abundances of *Actinobacteria*, *Gemmatimonadetes*, and *Chloroflexi* showed an increasing trend in the soil after planting dates.

Figure 5B illustrates the composition of the soil bacterial communities at the genus level, with the top 31 taxa obtained in terms of relative abundance. The top 10 taxa in terms of relative abundance were Acetobacter, Subgroup_6_unclassified, *Ralstonia*, MND1, RB41, *Rokubacteriales*_unclassified, WD2101_soil_group_unclassified, *Bacillus* spp. (*Gemmatimonadaceae*_unclassified), *Pedosphaeraceae*_unclassified, and *Actinobacteria* (*Gaiellales*_unclassified). These bacterial communities at the genus level generally aligned with the top 10 in terms of relative abundance at the phylum level.

#### 3.3.2. Soil Fungal Community Composition

**Figure 6:**
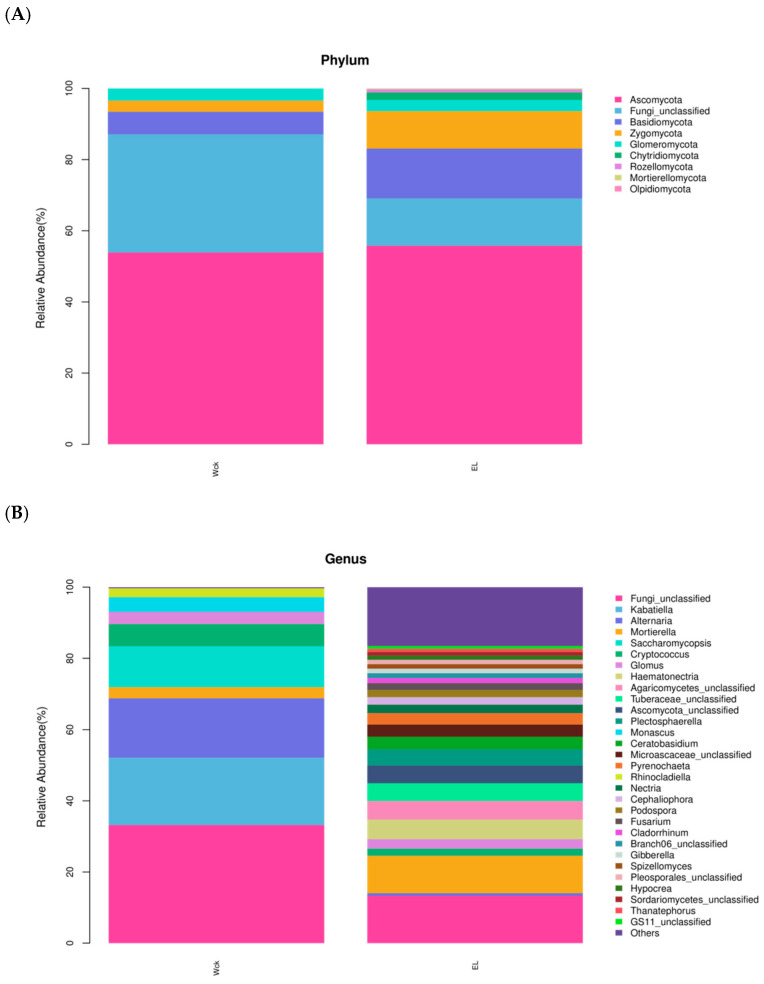
Abundance map of fungal communities at the phylum level (**A**) and genus level (**B**) in W and E. Note: W: Uncultivated soil with *E. angustifolia* L.; E: soil for planting *E. angustifolia* L.

Figure 6A illustrates the composition of the soil fungal communities at the phylum level, highlighting the nine most abundant taxa in terms of relative abundance. The fungal phyla with relative abundances greater than 1% were *Ascomycota*, Fungi_unclassified, *Basidiomycota*, *Zygomycota*, and *Glomeromycota*. *Chytridiomycota*, *Rozellomycota*, *Mortierellomycota*, and *Olpidiomycota* were not found in the bare soil control but were observed in the rhizosphere soil of the date palm. Additionally, the relative abundance of *Chytridiomycota* was higher than that of *Olpidiomycota*, above a threshold of 1%. The relative abundance of the five major phyla was greater in the inter-rhizosphere soil of dates than in soils without dates. Ascomycota was the dominant phylum in all samples, with a relative abundance greater than 25%. The relative abundance of unclassified fungal phyla was 2.49 times greater in the control soils than in the soils with dates. Furthermore, the relative abundances of *Proteobacteria*, *Joints*, and *Phaeophytes* were greater in the inter-rhizosphere soil with dates than in soils without dates.

Figure 6B depicts the composition of soil fungal communities at the genus level, highlighting the top 31 genera in terms of their relative abundance. The top 10 genera included Others, Fungi_unclassified, *Mortierella*, *Haematonectria*, *Agaricomycetes*_unclassified, *Tuberaceae*_unclassified, *Ascomycota*_unclassified, *Plectosphaerella*, *Ceratobasidium*, and *Microascaceae*_unclassified. Notably, the relative abundance of Others in the inter-rhizosphere soil of date palm was significantly higher than that in the control group. This study discovered that the relative abundance of Fungi_unclassified was 19.91% greater than that in the control group. Similarly, the relative abundance of *Mortierella* was 7.36% higher than that in bare soil. Furthermore, the relative abundances of *Haematonectria*, *Agaricomycetes*_unclassified, *Tuberaceae*_unclassified, and *Plectosphaerella* were 7.36% higher than those in the control group. Other notable taxa with increased relative abundances included unclassified, *Tuberaceae*_unclassified, *Ascomycota*_unclassified, *Plectosphaerella*, *Ceratobasidium*, *Microascaceae*_unclassified, and *Pyrenocephalus*. The relative abundance of all eight genera of *Pyrenochaeta* was greater in the rhizosphere soil than in the control soil, and the relative abundance of the control genera was close to 0%.

### 3.4. Correlation Between Soil Microorganisms and Soil Physicochemical Properties

#### 3.4.1. Relationships Between Microbial Phylum Levels and Soil Nutrients

**Figure 7:**
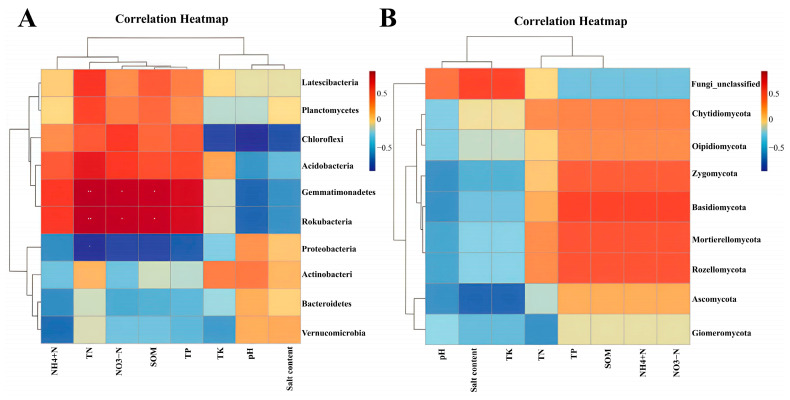
Heatmap of Spearman’s correlation between bacterial (**A**) and fungal (**B**) communities and soil properties. Note: W: Uncultivated soil with *E. angustifolia* L; E: soil for planting *E. angustifolia* L. ∗ Correlation is significant at the 0.05 level; ∗∗ correlation is significant at the 0.01 level.

As depicted in Figure 7A, the correlation analysis between soil bacterial communities at the phylum level and soil physicochemical properties revealed that soil physicochemical factors, such as soil TN, were significantly positively correlated with the phylum *Gemmatimonadetes* and the phylum *Rokubacteria*, and significantly negatively correlated with the phylum *Proteobacteria*. SOM and soil NO_3_^−^-N were significantly positively correlated with *Gemmatimonadetes* and *Rokubacteria* and negatively correlated with *Chloroflexi*. Soil TP, soil NH_4_^+^-N, soil TK, soil salinity (salt content), and soil pH were all negatively and positively correlated with the horizontal bacterial communities to varying degrees, while these correlations were not significant.

As depicted in Figure 7B, correlation analysis between soil fungal communities at the phylum level and soil physicochemical properties revealed that soil pH was positively correlated with Fungi_unclassified and negatively correlated with other fungal phyla. Conversely, soil TN was negatively correlated with Fungi_unclassified and positively correlated with other fungal phyla. Soil salinity (salt content) and soil TK were negatively correlated with *Ascomycota*, whereas these correlations were not significant. Soil TP, SOM, NH_4_^+^-N, and NO_3_^−^-N were negatively correlated with Fungi_unclassified and positively correlated with other fungal phyla.

#### 3.4.2. Relationship Between Microbial Phylum Levels and Soil Enzyme Activities

**Figure 8:**
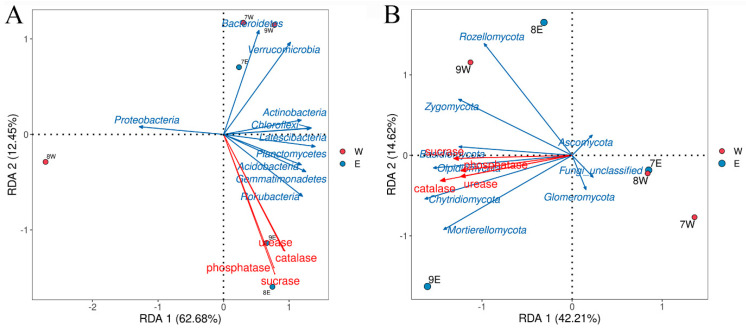
RDA was performed to analyse Spearman’s correlations between soil properties and bacterial communities (**A**) and fungal communities (**B**) at the phylum level, respectively. Note: W: Soil uncultivated with *E. angustifolia* L; E: soil for planting *E. angustifolia* L.

As depicted in Figure 8A, correlation analysis between soil bacterial communities at the phylum level and soil enzyme activities showed that the weight of both axes was 75.13% (RAD1 = 62.68%, RAD2 = 12.45%). The four soil enzyme activities were negatively correlated with *Proteobacteria* and positively correlated with other phyla, with sucrase activity playing the largest role. As shown in Figure 8B, correlation analysis between soil fungal communities at the phylum level and soil enzyme activities showed that the weight of both axes was 56.83% (RAD1 = 42.21%, RAD2 = 14.62%). The activities of the four enzymes were negatively correlated with *Ascomycota* and Fungi_unclassified, while *Glomeromycota* showed negative correlations. There were positive correlations with other phyla to varying degrees, with the greatest effect on peroxidase activity.

## 4. Discussion

The increase in SOM provides more energy and nutrients for soil microorganisms, promoting the circulation and transformation of nutrients in the soil, which is beneficial to the growth and development of plants. This study demonstrated that the content of SOM, TN, and TP significantly increased after the establishment of forests in *E. angustifolia* L., and the total salt content of the soil decreased significantly. This study observed a decrease in both soil salinity and pH. Previous research has demonstrated a covariant relationship between soil pH and salinity in saline soils during salinisation [23,24]. These results suggested that planting *E. angustifolia* L. can alleviate soil salinity and improve the soil environment. It is hypothesised that this effect can be attributed to soil dehydration, sedimentation, desalination, and decalcification. Additionally, SOM can also chelate some salts and release organic acids through decomposition to acidify the soil [25,26,27]. A neutral pH favours the utilisation of potassium. In contrast, both acidic and alkaline pH conditions can increase the total potassium (TK) content in the soil. The elevated pH levels in saline soils pose a constraint on the release of alkaline phosphatase. Moreover, the slow release of immobilised potassium can only take place when soil salinity decreases [28]. The combined effect of these factors has a beneficial ameliorative impact on soil properties and structure. It also supplements the soil with a nutrient source, which may account for the increase in TK content observed in this study. The primary source of variation in soil TP content is attributed to the presence of plant and microbial organophosphorus compounds. Organic phosphorus compounds in soil undergo hydrolysis reactions due to the action of soil microorganisms (belonging to *Firmicutes*, *Proteobacteria*, *Ascomycota*, *Actinobacteria*, etc.) and a variety of phosphatase enzymes secreted by plant roots [29]. These phosphatase enzymes can break down organic phosphorus into inorganic phosphates, such as H_2_PO_4_^−^ and HPO_4_^2−^, for plant utilisation [30]. The findings of this research indicated a noticeable upward trajectory in TP content. The results of the study showed a clear upward trend in TP. The observed increase in TP content may be due to phosphatases secreted by the plant root system to promote the fixation and accumulation of organic phosphorus under specific conditions, or it may be due to microbial uptake and utilisation of organic phosphorus to form organic phosphorus compounds in the body, which are then released into the soil when the microbes die, increasing the soil organic phosphorus content. In contrast, the inter-rhizosphere soil that was cultivated with dates exhibited notably elevated levels of organic matter and nutritional content compared with the control soil. Mineralisation involved the conversion of soluble organic nitrogen into NH_4_^+^-N by the action of organic matter. NH_4_^+^-N was then transformed into NO_3_^−^-N, which finally became organic nitrogen through biological absorption. As a result, the levels of TN and NO_3_^−^-N exhibited a gradual upward trend.

Soil enzymes, the most active organic components of soil, are primarily produced by microorganisms. They play a crucial role in the degradation, transformation, and mineralisation of organic matter, significantly affecting ecosystem processes [28]. Microbial activity in rice inter-rhizosphere soil has been identified to enhance soil enzyme activity and suppress pH [31]. The results of soil enzyme activity in this study showed that all four soil enzyme activities were significantly higher after planting dates than in non-date soils, consistent with previous studies. Many studies have shown that high soil organic matter content is associated with significantly higher soil urease activity that can reflect potential changes in soil N content [32]. According to Diard and Hardt, soil catalase activity correlates with soil respiration intensity and soil microbial activity, serving as an extremely important indicator of the soil microcosmic environment [33]. It can effectively mitigate the toxic effects of hydrogen peroxide. Phosphatase is an enzyme that dephosphorylates and produces phosphate ions and sucrase hydrolyses sucrose, thereby enhancing soil nutrients [34]. Soil enzymes are secreted by soil microorganisms, plants, and animal residues, in addition to being one of the main sources of soil microorganisms [35]. There is also a direct correlation between the soil enzyme activity and microorganisms.

In general, specific soil enzyme activities are closely associated with bacterial and fungal taxa. *Trichoderma* and *Pythium* elevate the activities of a variety of enzymes associated with the carbon (C), nitrogen (N), and phosphorus (P) cycles in sandy loam soils, such as acid and alkaline phosphatases, ureases, β-glucanases, and cellulolytic enzymes [36]. Meanwhile, bacteria such as *Azospirillum* and *Pseudomonas*, as well as the fungus *Trichoderma*, similarly enhanced soil enzyme activities [37,38]. The correlation between the four soil enzyme activities and the level of the bacterial phylum indicated a negative correlation with *Proteobacteria* and a positive correlation with the level of the other phyla, with sucrase activity playing the largest role. All four enzyme activities were negatively correlated with *Ascomycota* and *Glomeromycota*; they were positively correlated to various degrees with other phyla, with catalase activity being the largest. The abundance of soil microorganisms gradually increased after planting dates compared with that of the control, probably because the stimulation of inter-root secretions can produce new microbial communities associated with enzyme activity. In summary, the inter-rhizosphere abundance of date palms planted with date palm increased gradually compared with that of the control. Moreover, soil enzyme activity increased significantly in the inter-rooted soil after planting dates compared with that in the bare soil, indicating that planting dates had a more substantial impact on the soil microenvironment.

The microbial composition of the inter-rhizosphere soil of different plants can be relatively uniform and stable at the phylum level. Bacteria are mainly concentrated in the *Streptomyces*, *Actinobacteria*, *Bacteroidetes*, and *Thick-walled Bacteria* [39], and fungi are mainly concentrated in the phyla *Ascomycota* and genus *Aspergillus* [40]. This study aligned with previous findings, with the dominant bacterial phyla (*Streptomycetes*, *Acidobacteria*, *Actinobacteria*) in the inter-rhizosphere soil of *E. angustifolia* L. and the dominant fungal phyla of *Ascomycota*, and *Basidiomycota*. Compared with the control, there were significant fluctuations in the relative abundance of soil microbial communities at the phylum and genus levels in the inter-rhizosphere soil of *E. angustifolia* L. This suggested that the cultivation of *E. angustifolia* L. affected changes in the abundance and composition of the soil microbial communities. Research has shown a correlation between Amoebae and *Actinobacteria* and the suppression of diseases [41]. *Actinobacteria* have been found to facilitate plant development and contribute to disease management. Furthermore, it was shown that high abundance of salt-tolerant nitrogen-fixing endophytic bacteria at the genus level was associated with *Pseudomonas aeruginosa* in the roots of saline plants in saline soils [42].

Approximately 25% of plants inhabiting naturally nutrient-deficient soils rely on nitrogen-fixing bacteria, namely, rhizobacteria. These bacteria facilitate plant development by converting atmospheric nitrogen (N_2_) into ammonium (NH_4_^+^), which can be readily absorbed by plants. Nitrogen-fixing bacteria play a crucial role in providing nitrogen to the root system and improving soil fertility. Among these bacteria, *Proteobacteria* are particularly influential in nitrogen and energy cycling processes within soil ecosystems [43,44]. Therefore, increasing the soil diversity can significantly improve the soil quality and fertility. Studies have shown that thick-walled phyla and other classes, including *Bacillus*, are associated with plant promotion [45,46]. *Gemmatimonadetes* and *Bacteroidetes* prefer high-salinity soil environments because of their strong salinity adaptations [47]. SOM was significantly and positively correlated with both the Bacillus phylum (*Gemmatimonadetes*) and *Rokubacteria*, similar to the findings of Chi et al. [48]. The generation of soil organic carbon serves as a carbon source for soil microbial activity. This association may be attributed to the heightened microbial population and activity, which may be a consequence of the augmented input of plant-derived carbon sources.

The correlation study revealed a positive association between soil total nitrogen, total phosphorus, organic matter, and the green curvilinear phylum, which promoted increased soil nutrient content. Consequently, the eutrophic bacterial populations of *Actinobacteria*, *Bacillariophyta*, and *Bacillariophyta* grew abundantly and multiplied in high-nutrient soils, thereby increasing their abundance. Soil fertility is a vital factor affecting microbial communities. The fungal phyla *Cysticercus* and *Streptomyces* are important decomposers in soil. Most *Streptomyces* species are saprophytic and can decompose large quantities of the hard-to-degrade organic matter, thus increasing soil fertility [49]. This may be one of the reasons for the increase in soil nutrients after planting dates. Planting dates increase the effective nutrients in the soil, which increases the resources and space available for microorganisms in the soil and the environmental capacity, increasing the diversity of the microbial community [50].

## 5. Conclusions

In conclusion, cultivation of *E. angustifolia* L. has a significant impact on soil nutrient availability and microbial community structure. Compared with the inter-rhizosphere soil of planted *E. angustifolia* L. and unplanted *E. angustifolia* L., the nutrients in the soil were markedly enhanced following planting of *E. angustifolia* L. This was accompanied by changes in the diversity and structure of the inter-rhizosphere soil microbial community of *E. angustifolia* L. The abundance of soil microbial communities also increased, with the *Streptomycetes*, *Acidobacteria*, and the *Actinobacteria* in the bacterial group and the phyla *Ascomycota* and *Basidiomycota* in the fungal group demonstrating significant dominance. Furthermore, changes in soil organic matter, soil total phosphorus, and soil nitrogen, which are key physicochemical properties of the soil, have been identified as the primary factors affecting the composition of soil microbial communities.

## Data Availability

The data that support the findings of this study are openly available in the GenBank database with the primary accession code PV089868-PV090748, KIWZ00000000.

## Supplementary Materials

The following supporting information can be downloaded at: https://www.mdpi.com/article/10.3390/plants14081242/s1, Figure S1: Rarefaction curve of OTUS between bacterial (A) and fungal (B) communities in W and E.

## Abbreviations

*E. angustifolia* L.	*Elaeagnus angustifolia* Linn.
TN	Total nitrogen
NO_3_^−^-N	Nitrate nitrogen
NH_4_^+^-N	Ammonium nitrogen
TP	Total phosphorus
TK	Total potassium
SOM	Soil organic matter
N_2_	Atmospheric nitrogen
NH_4_^+^	Ammonium
