# Supplementary material for: Effect of Elaeagnus angustifolia Linn. on the Physicochemical Properties and Microbial Community Structure of Inter-Rhizosphere Soils
Source: Plants (Basel). 2025 Apr 18;14(8):1242. doi: 10.3390/plants14081242 (PMC12030227; doi:10.3390/plants14081242)
Supplement: Supplementary file 1 [file plants-14-01242-s001.zip › plants-3544339-supplementary.pdf]

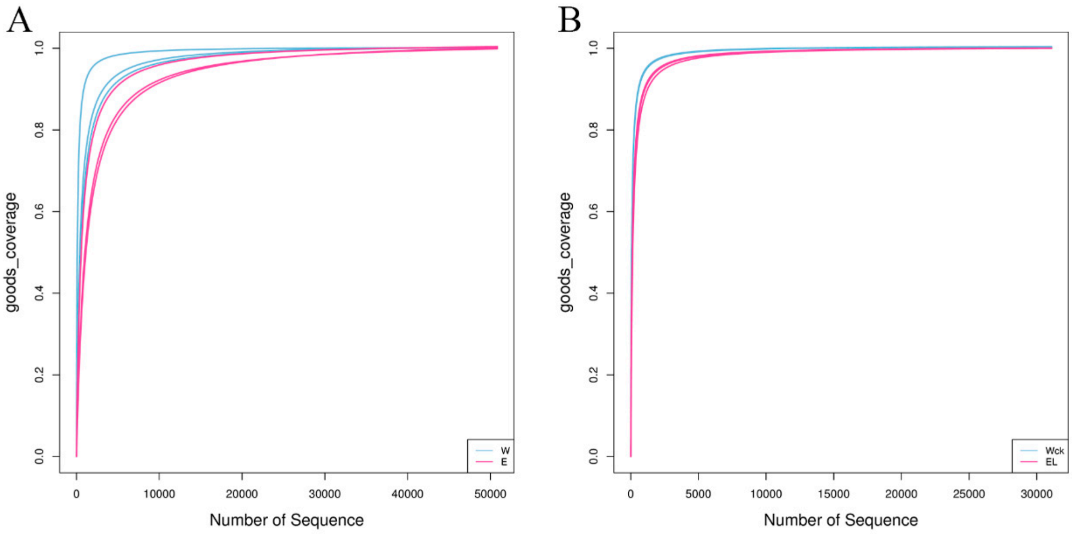

Figure S1**.** Rarefaction curve of OTUS between bacterial (A) and fungal (B) communities in W and E.

Goods _coverage is the microbial coverage, the higher the value, the lower the probability that a new species has not been detected in the sample, this index actually reflects whether the results of this sequencing represent the real situation of the sample.

Note: W: Uncultivated soil with *E. angustifolia* L.; E: Soil for planting *E. angustifolia* L.
